# Factors associated with delayed initiation of breastfeeding: a survey in Northern Uganda

**DOI:** 10.1080/16549716.2017.1410975

**Published:** 2017-12-15

**Authors:** David Mukunya, James K Tumwine, Victoria Nankabirwa, Grace Ndeezi, Isaac Odongo, Josephine Tumuhamye, Justin Bruno Tongun, Samuel Kizito, Agnes Napyo, Vincentina Achora, Beatrice Odongkara, Thorkild Tylleskar

**Affiliations:** ^a^ Center for International Health, University of Bergen, Bergen, Norway; ^b^ Department of Pediatrics and Child Health, Makerere University, Kampala, Uganda; ^c^ Department of Epidemiology and Biostatistics, School of Public Health, Makerere University, Kampala, Uganda; ^d^ Department of Pediatrics and Child Health, Makerere University, Kampala, Uganda; ^e^ Department of Pediatrics, University of Juba, Juba, South Sudan; ^f^ Clinical Epidemiology Unit, Makerere University, Kampala, Uganda; ^g^ Department of Public Health, Busitema University, Mbale, Uganda; ^h^ Department of Obstetrics and Gynecology, University of Gulu, Gulu, Uganda; ^i^ Department of Pediatrics, University of Gulu, Gulu, Uganda

**Keywords:** Nutrition, infant-feeding, post-conflict, breastmilk, neonatal

## Abstract

**Background**: Initiation of breastfeeding later than 1 hour after birth is associated with increased neonatal morbidity and mortality.

**Objective**: To determine the prevalence and factors associated with delayed initiation of breastfeeding.

**Methods**: We conducted a survey in 2016 of 930 children under the age of 2 years in Lira district, northern Uganda. Mothers of the children were interviewed and data was collected on mobile phones using Open Data Kit software (https://opendatakit.org). Multivariable logistic regression was used to determine factors associated with delayed initiation of breastfeeding.

**Results**: Almost half [48.2%, 95% confidence interval (CI) (44.3–52.1)] of the mothers delayed initiation of breastfeeding. Factors significantly associated with delayed initiation of breastfeeding in multivariable analysis included caesarean delivery [Adjusted Odds Ratio (AOR) 11.10 95% CI (3.73–33.04)], discarding initial breast milk [AOR 2.02 95% CI (1.41–2.88)], home delivery [AOR 1.43 95% CI (1.04–1.97)] and mother being responsible for initiating breastfeeding as compared to a health worker or relative [AOR 1.73 95% CI (1.33–2.26)]. Mothers having a secondary education were less likely [AOR 0.54 95% CI (0.30–0.96)] to delay initiation of breastfeeding as compared to those with no education.

**Conclusion**: About half the mothers delayed initiation of breastfeeding until after 1 hour after birth. Programs to promote, protect and support breastfeeding in this post conflict region are urgently needed.

## Background

Reduction of neonatal mortality to 12/1000 live births by the year 2030 is one of the targets of the third sustainable development goal (SDG) [1,2]. Sub-Saharan Africa has one of the highest neonatal mortality rates in the world at 28/1000 live births [3,4] with Uganda at 27/1000 live births [5]. Over 800,000 neonatal deaths annually could be prevented if breastfeeding practices were scaled up [6]. One of the important practices in scaling up breastfeeding is initiating breastfeeding within the first hour after birth, failure of which is termed delayed initiation of breastfeeding.

Delayed initiation of breastfeeding has been shown to increase the risk of neonatal morbidity and mortality [7–10]. The morbidity and mortality risk associated with delayed initiation of breastfeeding is due to a number of factors. Delayed initiation of breastfeeding increases the use of pre lacteal feeds, which are often contaminated, and also lack the mucosa protective effect of breast milk [7]. Newborns who are delayed breastfed are also deprived of an opportunity to fully benefit from the immunological protection offered by colostrum [7,9]. Another benefit that is lost when initiation of breastfeeding is delayed, is the body heat offered to the baby by the mother in the initial moments after birth [9]. Finally, mothers who delay the initiation of breastfeeding are more likely to use alternative or additional foods during the first 6 months of life [7,9]. Due to all the risks caused by delayed initiation, interventions that promote early initiation of breastfeeding are listed as priority intervention to improve neonatal health and survival [11].

The prevalence of mothers who delay initiation of breastfeeding is high both in Uganda [12] and the world at large [6] with only about half of mothers worldwide practicing early initiation of breastfeeding [6]. Factors associated with delayed initiation of breastfeeding include maternal-infant factors (such as absence of breast milk, HIV status of the parent and perinatal morbidity), cultural factors (such as colostrum discarding), and social factors (such as rural residence and place of delivery) among others [12–14]. However, breastfeeding initiation practices have been found to vary across ethnic lines and geographical locations [15]. This study was undertaken in post conflict Northern Uganda. Negative public health effects have been observed in post conflict areas lasting for so many years after the conflict [16]. The negative public health trends in post conflict areas are a result of poor health-seeking behaviors and distrust of health care systems among other reasons [17]. As such, public health interventions designed for post conflict areas might be of significant value.

To design interventions to scale up early initiation of breastfeeding, we need increased knowledge of associated factors. Therefore, in this community study, we aimed to determine the prevalence of delayed initiation of breastfeeding in Lira district, located in post conflict Northern Uganda and to determine factors associated with the delay.

## Methods

### Study design

This was a comparative cross-sectional study carried out among women with a child below the age of 2 years.

### Study setting

The study was conducted between August and November 2016 in Lira district, located in post conflict Northern Uganda. Lira district is home to about 400,000 people with the majority living in rural areas [18,19]. Most of the population is ethnic Langi and the predominant language spoken is Luo. Lira district has 3 Counties and 13 sub-counties, 1 municipality and 751 villages. Three sub counties of Aromo, Agali and Lira municipality were surveyed because they had been chosen as the sites for a randomized controlled trial designed to promote facility births and recommended breastfeeding practices. Aromo and Agali were chosen because they had the poorest maternal and child health indicators. Lira municipality was chosen because it was the largest urban center.

### Sampling

This study was a two-stage sampling modification of the WHO EPI method [20,21].

All villages in the three selected sub-counties and the household populations were listed (add range) and 30 villages were selected by probability proportional to size. In each village, a sampling frame of all households was then used to select a random index household. After identifying the first household, the next household was chosen by selecting the nearest household to the first (the one whose door was closest to the prior household). Only one mother-child pair would be chosen from each household. This process would continue until 31 mother-child pairs had been interviewed in each village.

### Study participants

We recruited mother-child pairs where the child was aged 2 years or less. Children were recruited only if they were residents in the village and were identifiable by the village leadership and were mentally and physically able to complete the interview. If the mother had more than 1 child born within the last 2 years, the youngest child was selected. If the mother had twins, she would answer child-related questions basing on the younger twin. Children with no mothers were excluded from the study.

### Study procedure

Thirty trained research assistants who were knowledgeable about the area and were fluent in both written and spoken forms of the local language collected data. Village health team members and local area leaders acted as community guides. Interviews were carried out at the mothers’ homesteads and where possible in a private location away from distraction within those homesteads. Thirty-one participants were recruited from each of the 31 villages. Missing households would be revisited later the same day before the research assistants left the village. Those that were not found at home were revisited the following day and if not found declared missing and replaced. Replacement was based on proximity to the missing household; the household with the nearest door to the missing household was visited and this process repeated until a replacement was got. Data were collected on mobile phones using ODK software (https://opendatakit.org).

### Data quality

We used a validated structured coded questionnaire, which was pretested during the piloting. The validated questionnaire was developed from two questionnaires; the first was a locally validated questionnaire from a breastfeeding promotion trial conducted in Eastern Uganda [22] and the second was an infant feeding assessment questionnaire developed by the World Health Organization [23]. A language expert translated the questionnaires to the local language and back translated them to ensure proper translation. The mobile data collection tool ensured completeness of the questionnaire using its checks, by preventing progression if a question had been left unanswered. Completed questionnaires were saved onto the mobile phone and uploaded onto a server at the end of each day. A data manager was available when needed to deal with any issues related to the mobile phone data collection process. To reduce the potential of a measurement bias, mothers were first asked about events surrounding the most recent pregnancy to refresh their memory. They were subsequently asked questions about the most recent delivery in the last 2 years as an effort to ease recall.

### Variables

The dependent variable was initiation of breastfeeding. Women were asked how long after birth the baby was put to the breast for the first time. Responses were recorded in minutes and/or hours. The dependent variable was categorized as early initiation if breastfeeding was initiated within the first hour after birth and late initiation if breastfeeding was initiated later than 1 hour after birth. Exposure variables (Tables 1–3) included mother’s age at last birthday collected as a continuous variable but categorized into <19, 20–24, 25–29, 30–34, and ≥35. Maternal and paternal education was categorized as none, primary, secondary and tertiary education. Mother’s employment (activity outside the home) was categorized as yes or no, Marital status was categorized as single if the mother was not living with a partner (single, divorced, widowed, separated) and married if the mother was living with a partner (married, cohabiting). Residence was categorized as (rural/urban). Other variables included; parity (1, 2 or 3, 4, >5), place of delivery (facility/home), mode of delivery (vaginal/caesarean delivery), any complications during delivery such as; vaginal bleeding, obstructed labor, postpartum hemorrhage, sepsis, birth asphyxia, cord prolapse, small baby, premature baby (yes/no), breathing/crying problem at birth (yes/no), prematurity at birth (yes/no), singleton versus multiple births, sex of the child (male/female), placement of baby immediately after delivery (side of mother, abdomen or chest of mother, other), receipt of breastfeeding counseling during pregnancy (yes/no), person responsible for initiation of breastfeeding (mother/person other than mother) and discard (throwing away) of initial breast milk (yes/no).

**Table 1.** Baseline characteristics of mother-infant pairs surveyed in Northern Uganda.

	All participants	Delayed initiation of breastfeeding
Variable	N = 930n (%)	N = 448n (%)
Sex of the child		
Male	465 (50.0)	231 (51.6)
Female	465 (50.0)	217 (48.4)
Mother age		
≤19	157 (16.9)	84 (18.8)
20–24	303 (32.6)	138 (30.8)
25–29	218 (23.4)	97 (21.7)
30–34	145 (15.6)	70 (15.6)
≥35	107 (11.5)	59 (13.2)
Mothers education		
None	102 (11.0)	56 (12.5)
Primary	729 (78.4)	355 (79.2)
Secondary	83 (08.9)	29 (06.5)
Tertiary	16 (01.7)	8 (01.8)
Paternal education		
None	24 (02.8)	11 (02.7)
Primary	524 (61.4)	261 (63.5)
Secondary	228 (26.7)	107 (26.0)
Tertiary	77 (09.0)	32 (07.8)
Marital status		
Single	77 (8.3)	37 (08.3)
Married	853 (91.7)	411 (91.7)
Maternal employment		
No	537 (62.0)	266 (63.9)
Yes	329 (38.0)	156 (36.1)
Parity		
1	227 (24.4)	114 (25.5)
2–3	298 (32.0)	139 (31.0)
4>	405 (43.6)	195 (43.5)
Residence		
Rural	589 (63.3)	290 (64.7)
Urban	341 (36.7)	158 (35.3)

**Table 2.** Delivery characteristics of mother-infant pairs surveyed in northern Uganda.

	All participants	Delayed initiators of breastfeeding
Variable	N = 930n (%)	N = 448n (%)
Mode of delivery		
Vaginal	894 (96.1)	416 (92.9)
Caesarean	36 (03.9)	32 (07.1)
Place of delivery		
Facility	622 (66.9)	284 (63.4)
Home	308 (33.1)	164 (36.6)
Breathing/crying problem		
No	816 (87.7)	384 (85.7)
Yes	114 (12.3)	64 (14.3)
Discarded initial milk		
No	675 (72.6)	296 (66.1)
Yes	255 (27.4)	152 (33.9)
Advised on breastfeeding during pregnancy		
No	379 (40.8)	193 (43.1)
Yes	551 (59.3)	255 (56.9)
Complications during birth		
No	820 (88.2)	380 (84.8)
Yes	110 (11.8)	68 (15.2)
Place baby initially put		
Other	82 (08.8)	51 (11.4)
Stomach/chest of mother	563 (60.5)	255 (31.0)
Side of mother	285 (30.7)	142 (31.7)
Baby born at term		
No	49 (05.3)	23 (05.1)
Yes	881 (94.7)	425 (94.9)
Who was responsible for initiation		
Other	431 (46.3)	188 (42.0)
Mother	499 (53.7)	260 (58.0)

**Table 3.** Factors associated with delayed initiation of breastfeeding at bi-variable and multivariable analysis among mother-infant pairs in Northern Uganda.

	Bi-variable N = 930		Multivariable N = 930	**Multivariable N = 866
Variable	OR (95% CI)	p value	AOR (95% CI)	AOR (95% CI)
Sex of the child				
Male n (%)	1	0.342	-	-
Female n (%)	0.89 (0.69–1.14)			
Mothers education				
None	1	0.281	0.87 (0.53–1.43)	0.87 (0.54–1.40)
Primary	0.78 (0.49–1.24)	0.007	0.54 (0.30–0.96)	0.50 (0.29–0.85)
Secondary	0.44 (0.25–0.79)	0.758	0.80 (0.18–3.56)	0.83 (0.19–3.57)
Tertiary	0.82 (0.23–2.99)			
Marital status (living with partner)				
Single	1	0.983	-	-
Married	1.00 (0.61–1.65)			
Parity				
1	1	0.424	-	-
2–3	0.87 (0.60–1.24)	0.645		
4>	0.92 (0.64–1.33)			
Residence				
Urban	1	0.449	-	-
Rural	1.12 (0.82–1.53)			
Mode of delivery				
Vaginal	1	0.001	11.10 (3.73–33.04)	10.09 (3.35–30.43)
Caesarean	9.19 (2.76–30.66)			
Place of delivery				
Facility	1	0.051	1.43 (1.04–1.97)	1.49 (1.09–2.03)
Home	1.36 (1.00–1.84)			
Breathing/crying problem at birth				
No	1	0.089	1.42 (0.92–2.20)	1.41 (0.91–2.17)
Yes	1.44 (0.94–2.20)			
Discarded initial milk				
No	1	0.001	2.02 (1.41–2.88)	-
Yes	1.89 (1.35–2.65)			
Advised on breastfeeding				
No	1	0.243	-	-
Yes	0.83 (0.60–1.14)			
Complication during birth				
No	1	0.004	1.30 (0.79–2.12)	1.29 (0.79–2.12)
Yes	1.87 (1.24–2.84)			
Place baby initially put				
Stomach/chest of mother	1	0.226	-	-
Side of mother	1.20 (0.89–1.62)	0.021		
Other place	1.99 (1.12–3.53)			
Baby born at term				
No	1	0.869	-	-
Yes	1.05 (0.55–2.00)			
Who was responsible for initiation of breastfeeding?				
Person other than mother	1	0.008	1.73 (1.33–2.26)	1.61 (1.25–2.07)
Mother	1.41 (1.10–1.79)			

**Multivariable: Model without ‘discarding of initial milk variable’ -: Excluded from multivariable model

### Sample size estimation

A total of 930 mother-child pairs were enrolled in the study. This was calculated by Open-Epi (*http://www.openepi.com*) assuming a prevalence of 51% who practice early initiation of breastfeeding, a prevalence obtained in a community study done in eastern Uganda [24]. We assumed a precision of 5%, and a design effect of 2. This gave us a sample size of 768 participants. Assuming a non-response of 15% we came up with a sample size of 904. To achieve a self-weighted sample, we decided to enroll 31 children from each of the 30 villages.

About factors associated with delayed initiation of breastfeeding, we calculated sample sizes of various exposures and finally used ‘place of delivery’ which gave us the largest sample size. We calculated a sample size needed to detect differences in the proportion of delayed initiation of breastfeeding between mothers who delivered at home and those who delivered from the facility (place of delivery resulted in the largest sample size among the factors associated with delayed initiation of breastfeeding). We calculated this using Open-Epi [25] sample size calculation for detecting differences between proportions of two groups (Fleiss with CC) assuming that 60% of mothers delivered in health facility whereas 40% delivered from home, and assuming the proportion of delayed initiation being 70% for those who delivered at home and 58% for those who delivered at a health facility [26]. This yielded a sample size of 551, which was covered by the sample size calculated for the prevalence estimate.

### Data analysis

We used Stata version 14 (*http://www.stata.com/stata14/*) with survey set command adjusting for the multistage sampling in the analysis. Continuous descriptive variables were presented as means and standard deviations. Categorical variables were presented as proportions. We used chi square tests to tests for comparison of categorical variables and reported the resultant p values. We performed bi variable and multivariable logistic regression to determine the association between the independent factors and delayed initiation of breastfeeding. Factors known to be predictors of delayed initiation of breastfeeding from the literature and those with a bi-variable p-value <0.25 (as long as they were not in the casual pathway and they were not strongly collinear with other independent variables) were considered for the initial multivariable model [27]. Collinearity was assessed and factors were considered to be strongly collinear if their variance inflation factor was greater than 10. In case of collinearity, the factor with a stronger measure of association with the outcome variable was retained and the other dropped. Variables considered for the initial multivariable model were entered in a backward stepwise logistic model, and the variables that were dropped were assessed for their confounding effect on a model that only had the factors retained in the stepwise model. A variable was called a confounder if it changed the unadjusted measure of association by 10% or more. The final model included all confounders and was tested for goodness of fit using the Hosmer and Lemeshow goodness of fit test [28]. The process of multivariable modeling was repeated without the variable ‘discarding of initial milk’.

## Results

### Baseline characteristics

A total of 930 mother-child pairs were included in the analysis. The response rate was 93%. The majority (>95%) of non-respondents were mothers who were absent from their homestead. The mean (standard deviation, SD) child age was 11.2 (7.7) months. The mean (SD) maternal age was 25.8 (5.9) years. Most mothers and fathers had only primary education. The majority of the women had no employment and 44% had so far given birth to 4 or more children (Tables 1 and 2).

### Bivariable analysis

Almost a half [48.2%, 95% Confidence Interval (CI) (44.3–52.1)] of mothers delayed initiation of breastfeeding. When analysis was restricted to only those with children aged 1 month or less (n = 87), the proportion of mothers who delayed initiation of breastfeeding was 55.2%, 95% CI (44.3–65.6). Among mothers with children aged 3 months or less (n = 172), 50.6% of the mothers delayed initiation of breastfeeding, 95% CI (42.9–58.2). The factors associated with delayed initiation of breastfeeding in bi-variable analysis were; placing the baby away from the mother, having a caesarean section, discarding of initial milk, complications during delivery, and mother being solely responsible for initiation of breastfeeding (Table 3). Of the women who delivered from home, the mother was solely responsible for initiating breastfeeding 55% (n = 171) of the time. Mothers who delivered by caesarean section were significantly more likely to receive help from other persons to initiate breastfeeding compared to those who delivered vaginally [78% (n = 28) versus 45% (n = 403) respectively].

### Multivariable analysis

The factors significantly associated with delayed breastfeeding initiation at multivariable analysis included caesarean delivery Adjusted Odds Ratio (AOR) 11.10 95% CI (3.73–33.04), discarding initial breast milk AOR 2.02 95% CI (1.41–2.88), home delivery AOR 1.43 95% CI (1.04–1.97) and mother being solely responsible for initiating breastfeeding AOR 1.73 95% CI (1.33–2.26). Having a secondary education was protective against delayed initiation of breastfeeding when compared to no education [AOR 0.54 95%CI (0.30–0.96)] (Table 3).

In a model without discarding initial milk, the same factors above were associated with delayed initiation of breastfeeding and the measures of association were similar.

## Discussion

In this community-based survey conducted in post conflict northern Uganda, almost half of all mothers initiated breastfeeding later than an hour post-partum. Factors associated with this delay included caesarean delivery, discarding initial breast milk and the mother being responsible for initiating breastfeeding. Mothers who had a secondary education were less likely to initiate breastfeeding after 1 hour.

The proportion of mothers who delayed initiation is similar to findings from other African countries [6]. Analysis of the demographic health survey data showed almost half of women in Uganda delay to initiate breastfeeding [6]. Another community survey done in Eastern Uganda showed that only 51% of mothers initiated breastfeeding immediately after birth [24]. This proportion is, however, different from another study done at the national referral hospital, which found a much higher proportion of almost 70%, of mothers initiating breastfeeding early [12]. The difference could be explained by the policies at the national referral hospital or differences in the populations studied.

One of the major factors associated with delay in initiating breastfeeding was the practice of discarding the initial milk (colostrum). This practice has also been observed in Ethiopia [29] and Guatemala [30]. Another study conducted in Guinea-Bissau also noted that negative cultural ideas about colostrum were associated with delayed initiation of breastfeeding [31]. The practice of discarding colostrum is often due to a misconception that the initial milk is dirty and hence harmful to the neonate [31]. Mothers with this practice will probably wait for the milk to clear before they initiate breastfeeding and hence the delay. However, there is also a reverse causation possibility; that the practice of discarding initial milk was a result of delayed breastfeeding initiation linked to other factors like neonatal morbidity. The mother might be left with no other option but to express and discard the initial milk to alleviate the discomfort caused by engorged breasts. In such a scenario, discarding initial milk would be a consequence and not a cause of delayed initiation of breastfeeding. To control for this scenario, we repeated the multivariable modeling without the variable of ‘discarding initial milk’ but we found similar results.

In addition to discarding initial milk, delivering from home was also associated with delayed breastfeeding initiation. Mothers who delivered at home were more likely to delay initiating breastfeeding compared to mothers who delivered from health facilities. Similar findings have been reported in Nigeria [26]. This could be related to the existing policies and assistance offered in some health facilities, which encourage early initiation of breastfeeding [32]. To further support the hypothesis that mothers needed assistance to initiate breastfeeding, mothers who were solely responsible for initiating breastfeeding delayed in comparison to the mothers who reported receiving assistance from health workers or relatives. The importance of persons other than the mother in decisions related to breastfeeding practice, and the need to train them for this role is one of the tenets affirmed by the academy of breastfeeding medicine [32]. In addition, breastfeeding consultants at facilities medicine also advocates for a special position of a breastfeeding expert who among other roles assists caregivers and other health workers make decisions concerning breastfeeding [32–34].

Among those that delivered from health facilities, caesarean section delivery was the main factor associated with delayed initiation of breastfeeding. This association has also been observed in other studies done in Uganda, Nigeria and Vietnam [12,26,35]. The practice of separating the newborn from the mother immediately after caesarean delivery could partly explain this delay [36]. In addition, encouraging women to initiate breastfeeding early after caesarean section is uncommon [12,36,37]. Fatigue and post caesarean pain may also contribute to this delay [12]. Specific support for early initiation of breastfeeding is needed for mothers who deliver by caesarean section in order to reduce the delay in this group of women [37]. In this population, only 4% of women delivered by caesarean section, and this makes the relative contribution of this variable appear small. However, the caesarean section rate in this area is much lower than the minimum rate needed for optimal maternal and child health [38]. This suggests that the low cesarean rate is probably a result of poor health systems and therefore will become higher as the health systems in the area recover.

Lastly, education was another factor associated with delayed initiation of breastfeeding. Mothers with a secondary education were less likely to delay initiating breastfeeding. This pattern was also observed in a similar study done in Nepal [39], which showed that educated mothers were less likely to delay initiation of breastfeeding. Educated mothers have been shown to feed their children with more attention, discipline, purpose, and intentionality, and this could explain the association observed [40].

### Strengths and limitations

Our study assessed breastfeeding patterns from the community and is therefore more generalizable than prior hospital-based studies. Conducting a community-based study enabled us to capture and comment about home deliveries, which are common in this setting. In addition, the study looked at a unique setting, which is post conflict northern Uganda. A major limitation of our study was measurement of the main outcome with a 2-year recall window. The long recall periods of the time to initiating breastfeeding might have introduced a misclassification bias [41–43]. We used this long recall window mainly to enable us obtain sufficient mothers to study breastfeeding initiation in the community using multistage sampling. To gauge the magnitude of the potential misclassification we conducted sub group prevalence estimates for mothers who had children under 1 month, 3 months and under 2 years. We found these estimates to be 55%, 50% and 48%, respectively. From these estimates, we note that there was no major difference in recall for this variable based on time. A validation study done in Eastern Uganda showed high consistency between infant feeding questions asked at birth and those asked at 3 months [44], further research is needed to assess the consistency after 1 year or more. That said, a 2-year recall period is the recommended standard by the World Health Organization [23] and is also the standard used by the demographic and Health surveys which makes it a meaningful period to use for comparative purposes.

## Conclusion

About half of the mothers delayed initiation of breastfeeding until after 1 hour after birth. Programs to promote, protect and support breastfeeding in this post conflict region are urgently needed. We recommend that attention should be paid to caesarean section deliveries, and special policies concerning breastfeeding initiation in this group be considered. Persons other than the mother should be involved in programs promoting breastfeeding initiation.

## Paper context

Breastfeeding initiation patterns vary across different contexts. This study was done in Lira district, situated in post conflict northern Uganda, to inform the conduct of a randomized controlled study. Our study showed that delayed initiation is common in this region associated with factors such as caesarean delivery, home delivery, lack of education and discarding of initial milk. This study also showed that importance of persons other than the mother in initiating breastfeeding.
